# New Orleans Dental College

**Published:** 1870-04

**Authors:** 


### NEW ORLEANS DENTAL COLLEGE.

The annual commencement exercises of this institution took place on Thursday, the 10th inst., at the rooms of the college, corner of Carondelet and Perdido streets.

A large and appreciative audience was present, among which were many ladies.

After the Dean had announced the order of exercises, Prof. J. S. Harrison, Lecturer on Materia Medica and Therapeutics, delivered a very interesting and appropriate farewell address on behalf of the Faculty.

The Dean, Prof. J. S. Knapp, conferred the degree of Doctor of Dental Surgery, by virtue of the authority vested in him, accompanying the ceremony with a few excellent remarks, on five students who had conformed to the requirements for graduation.

Twenty-three honorary degrees were also awarded to dentists of long experience, eminence and merit, as skillful operators, residing in several of the Southern States.

An excellent valedictory was delivered on behalf of the graduating class by Dr. J. B. Wasson, of Memphis, Tenn.

The exercises were varied with music from a fine band, engaged for the occasion.

This institution, though organized in 1867, is in a flourishing condition, and is well supported by a corps of able teachers.

A free Dental Dispensary will be maintained, where poor people can be operated upon gratuitously every day, from 3 to 5 o'clock, at 67 Carondelet street.

